# Family-based association study of the BDNF, COMT and serotonin transporter genes and DSM-IV bipolar-I disorder in children

**DOI:** 10.1186/1471-244X-9-2

**Published:** 2009-02-04

**Authors:** Eric Mick, Janet Wozniak, Timothy E Wilens, Joseph Biederman, Stephen V Faraone

**Affiliations:** 1 Department of Psychiatry, Massachusetts General Hospital and Harvard Medical School, Boston, MA, USA; 2 Department of Psychiatry, SUNY Upstate Medical University, Syracuse, NY, USA; 3 Department of Neuroscience and Physiology, SUNY Upstate Medical University, Syracuse, NY, USA

## Abstract

**Background:**

Over the past decade pediatric bipolar disorder has gained recognition as a potentially more severe and heritable form of the disorder. In this report we test for association with genes coding brain-derived neurotrophic factor (*BDNF*), the serotonin transporter (*SLC6A4*), and catechol-O-methyltransferase (*COMT*).

**Methods:**

Bipolar-I affected offspring triads (N = 173) were drawn from 522 individuals with 2 parents in 332 nuclear families recruited for genetic studies of pediatric psychopathology at the Clinical and Research Program in Pediatric Psychopharmacology and Adult ADHD at Massachusetts General Hospital.

**Results:**

We failed to identify an association with the val66 allele in BDNF (OR = 1.23, p = 0.36), the COMT-l allele (OR = 1.27, p = 0.1), or the HTTLPR short allele (OR = 0.87, p = 0.38).

**Conclusion:**

Our study suggests that the markers examined thus far in *COMT *and *SLC6A4 *are not associated with pediatric bipolar disorder and that if the val66met marker in *BDNF *is associated with pediatric bipolar disorder the magnitude of the association is much smaller than first reported.

## Background

Several lines of evidence suggest that the age at onset of bipolar disorder may be a potentially important marker of a more severe and more familial form of the disorder. Consistent with the clinical observations made by Carlson et al [1] 25 years ago, the current literature suggests that pediatric bipolar disorder is associated with severe impairment, rapid cycling, early age at onset and significant psychiatric comorbidity. The presentation of bipolar disorder in children is not unlike that described in adults as mixed mania by McElroy et al [2]: a rapid-cycling and recurrent disorder with poor inter-episode functioning, frequent onset in childhood and adolescence, a high risk of suicide, high risk of substance use disorders, poor response to treatment, a history of poor school performance, and neuropsychological deficits highly suggestive of attention-deficit/hyperactivity disorder [3-11].

Studies of adult subjects with bipolar disorder strongly suggest a genetic component in etiology of the disorder. Twin studies [12-17] suggest heritability estimates from 79% to 93% for mania/bipolar disorder. Smoller and Finn [18] estimated a 10-fold increase in the recurrence risk (8.7%) for bipolar disorder among first-degree relatives of bipolar probands. Likewise, an excess recurrence risk of bipolar disorder has been reported in family studies of affected child probands utilizing DSM-III [19-22], DSM-IIIR [23], and DSM-IV [6,8,10,24] criteria for bipolar disorder. The recurrence risks reported in the pediatric literature (12–35%) are considerably higher than estimates of the risk for bipolar disorder in the relatives of adult probands (5–10%) [18,25].

Despite the evidence documenting that early-onset of bipolar disorder increases the familial recurrence rate, there has been very little molecular genetic research focused on pediatric bipolar disorder. Each of the candidate genes examined for association with pediatric bipolar disorder were selected largely because they are involved with neurotransmitter systems hypothesized to be altered in mood disorders based upon pharmacological response or animal models. However, only five genes have been examined and four of the available reports come from a single sample of approximately 50 families at Washington University [26-29].

The serotonin transporter is a monoamine transporter protein that transports serotonin from synaptic spaces into presynaptic neurons. A 44-bp variable-number tandem-repeat (VNTR) polymorphism in the in the promoter region (HTTLPR) of the serotonin transporter gene (*SLC6A4*) influences the transcription and function of the transporter [30]. Geller et al [26] found no evidence of association with the serotonin transporter gene (*SLC6A4*) at the HTTLPR locus (i.e. a 44-bp VNTR in the promoter) in 46 affected offspring trios.

Catechol-O-methyltransferase (COMT) catalyzes the transfer of a methyl group from S-adenosylmethionine to catecholamines, including the neurotransmitters dopamine, epinephrine, and norepinephrine [31]. A variant caused by a G-to-A transition at codon 158 of the COMT gene, results in a valine-to-methionine substitution [32]. Homozygosity for 158met leads to a 3- to 4-fold reduction in enzymatic activity, compared with homozygosity for 158val [32] but this marker was also not associated with bipolar disorder in 52 affected offspring trios [27].

Brain-derived neurotrophic factor (BDNF) supports survival of central nervous system neurons and stimulates growth and differentiation of developing neurons [33]. A polymorphism producing an amino acid substitution (valine to methionine) at codon 66 of the BDNF gene may impact intracellular trafficking and activity-dependent secretion of BDNF [34]. Geller et al [28] observed a significant over-transmission of the Val66 allele in 53 affected offspring trios of children with pediatric bipolar disorder.

The glutamate decarboxylase 1 gene (GAD1) is expressed in the brain and codes GAD67 which catalyzes the decarboxylation of glutamate to GABA [35,36]. Geller et al [29] examined the association with a SNP (rs2241165) that tags an over-transmitted haplotype of GAD1 associated with childhood schizophrenia [37] and found that the A allele was also significantly associated with pediatric bipolar disorder.

The dopamine transporter (DAT) plays a central role in regulation of dopaminergic neurotransmission via reuptake of synaptic dopamine [38,39]. We previously conducted a family-based association study of dopamine transporter gene (*SLC6A3*) in 170 affected offspring trios defined by a child (12.9 ± 5.3 years of age) with DSM-IV Bipolar-I disorder and found a positive association with one HapMap SNP (rs40184) that survived correction for multiple statistical comparisons (p = 0.038) [40].

In the current report we test for association with the *BDNF *(Val66Met), *COMT *(Val158Met), and *SLC6A4 *(HTTLPR) genes in these families in an attempt to replicate the findings previously reported by Geller et al [26-28]. We were not aware of the association with *GAD1 *[29] when candidates were being genotyped so cannot report results for that gene. We expected to find over-transmission of the *BDNF *val66 allele but no evidence of association with makers in either *COMT *or *SLC6A4*.

## Methods

Bipolar-I affected offspring triads (N = 173) were drawn from 522 individuals with 2 parents in 332 nuclear families recruited for genetic studies of pediatric psychopathology at the Clinical and Research Program in Pediatric Psychopharmacology and Adult ADHD at Massachusetts General Hospital [41-44]. All studies were sampled from the same source population and used the same assessment methodology regardless of the disorder used to classify subjects as cases. All study procedures were reviewed and approved by the Partners Healthcare Human Research Committee. All subjects' parents or guardians signed written informed consent forms and children older than 7 years of age signed written assent forms.

All affected offspring in the current analysis were diagnosed with bipolar I disorder according to DSM-IV criteria. The DSM-IV requires subjects to meet criterion A for a distinct period of extreme and persistently elevated, expansive or irritable mood lasting at least one week, plus criterion B, manifested by three (four if the mood is irritable only) of seven symptoms during the period of mood disturbance. Also recorded was the onset of first episode, the number of episodes, offset of last episode, and total duration of illness. Psychiatric assessments of child family members were made with the KSADSE (Epidemiologic Version) [45] and assessments of adult family members were made with the Structured Clinical Interview for DSM-IV [46]. Diagnoses were based on independent interviews with mothers and direct interviews with the children older than 12 years of age. Data were combined such that endorsement by either reporter resulted in a positive diagnosis. A committee of three psychiatrists, each board certified in both child and adult psychiatry, resolved all diagnostic uncertainties. The committee members were blind to the subjects' ascertainment group, ascertainment site, and data collected from family members.

All genotyping was conducted at the Psychiatric and Neurodevelopmental Genetics Unit of the Massachusetts General Hospital. Lab technicians were not aware of the source of the samples and all genotyping was performed in duplicate. The genotyping for SNPs in *COMT *(rs4680) and *BDNF *(rs6265) was performed using a single base extension reaction with allele discrimination by MassArray mass spectrometry system (Bruker-Sequenom) as previously described [47]. Genotyping of the SLC6A4 promoter (5-HTTLPR) polymorphisms was performed using the following protocol. Genomic DNA (5 ng) was amplified in a 7 μl reaction using KlenTaq DNA Polymerase (0.2 U), the proprietary KlenTaq Buffer (1×), dNTPs (200 μM each), 5% glycerol, Betaine (1 M) and the marker specific primers (0.2 μM). The SLC6A4 promoter VNTR primers were as follows: SLC6A4_PRO-F 6FAM-ATGCCAGCACCTAACCCCTAATGT, SLC6A4_PRO-R GGACCGCAAGGTGGGCGGGA. Amplification was performed with the following protocol: thirteen cycles of denaturation for 30 seconds at 93°C, annealing for 30 seconds beginning at 61.5°C and dropped 0.5°C every cycle and primer extension at 72°C for 30 second; 37 cycles of denaturation for 30 seconds at 93°C, annealing for 30 seconds at 55°C and primer extension at 72°C for 30; 72°C for 1 hour. Amplified products were pooled and combined with size standard (LIZ-250) before being analyzed on an ABI-3730. GeneMapper v3.5 was used to analyze the raw results from the ABI3730, however, a genotype was not considered final until two laboratory personnel had independently checked (and corrected) the GeneMapper results and both individuals were in agreement.

For the Transmission Disequilibrium Test (TDT) we used the PLINK (v1.0) software package [48]. Findings were considered statistically significant at p < 0.05.

## Results

Bipolar offspring were 12.8 ± 5.2 years old at assessment and predominantly male (74%, N = 127). As described above, all children met criteria for DSM-IV bipolar I disorder. The mean age at onset was 7.5 ± 4.7 years of age and the clinical presentation was chronic (3.5 ± 3.8 years duration), with rapid cycling (22.4 ± 82.3 episodes), and functional impairment (GAF score of 41.0 ± 6.1).

"Risk" alleles, designated to coincide with the previous reports of Geller et al, were in Hardy-Weinberg equilibrium for each gene assessed: *BDNF *(Val66, HWE p = 0.99), *COMT *(COMT-l allele, HWE p = 0.91), *and SLC6A4 *(HTTLPR short allele, HWE p = 0.72). Allele frequencies and association results are presented in Table 1. Seventy-six parents were heterozygous for the BDNF Val66 allele, 142 parents were heterozygous for the COMT-l allele, and 146 parents were heterozygous for the SLC6A4 short allele. We failed to identify significant associations with any of these candidates.

**Table 1:** TDT Analysis of candidate genes for pediatric bipolar disorder

**Gene**	**Marker**	**Allele^1^**	**Frequency**	**T:U^2^**	**OR**	**95% CI**	**χ^2^_(1)_**	**p-value**
BDNF	Val66Met	Val66	0.85	43:35	1.23	0.79–1.92	0.8	0.37
COMT	Val158Met	COMT-l	0.50	85:67	1.27	0.92–1.75	2.1	0.14
SLC6A4	HTTLPR	Short	0.46	73:84	0.87	0.63–1.19	0.8	0.38

**T:U **(trasmitted:untransmitted counts from basic TDT); **OR 95% CI **(odds ratio and 95% confidence interval from basic TDT. ^1 ^Val66 (G allele at rs6265), COMT-L (Met158; A allele at rs4680), Short (14-repeat allele). ^2 ^76 parents were heterozygous for the BDNF Val66 allele, 142 parents were heterozygous for the COMT-l allele, and 146 parents were heterozygous for the SLC6A4 short allele.

## Discussion

In this relatively large sample of families with a child proband diagnosed with DSM-IV bipolar-I disorder, we replicated earlier reports of no association with the disorder and *COMT *and *SLC6A4 *(SERT) [26,27]. We did not replicate the prior association with *BDNF *[28]. Geller et al reported a statistically significant (FBAT p-value 0.014) association in which the Val66 allele was transmitted 21 times and not transmitted 9 times (OR = 2.3, 95% CI = 1.09–4.98). We should have been able to detect an association of the magnitude observed in Geller et al [28]: the power to detect an odds ratio of 2.5 in our data was 0.89 at p < 0.05. If we pool the number of transmitted and not transmitted alleles in our data with that of Geller et al [28], the original estimate of association is attenuated and no longer statistically significant (OR = 1.45, 95% CI = 0.99–2.1, p = 0.1). Of course, a study of 170 families is modest for genetic association studies and the sample size needed to detect an odds ratio of 1.5 at p < 0.05 is 562 trios.

Despite the lack of replication observed in our data, *BDNF *may continue to be an interesting candidate gene for pediatric bipolar disorder. Although it was not found to be associated with bipolar disorder in genome-wide association studies of the disorder [49-52], Sklar et al [47] found association with *BDNF *in a large candidate gene family study of adults with bipolar disorder. Although preliminary, a recent study suggests that decreased BDNF gene expression may play a role in the pathophysiology of bipolar disorder in children. Pandey et al. [53] observed significantly lower BDNF mRNA levels in lymphocytes of children with bipolar disorder compared to healthy control children. Furthermore, treatment with mood stabilizers normalized BDNF mRNA level at 8 weeks and change in symptoms of bipolar disorder was correlated with change in BDNF mRNA levels [53].

Our results should be interpreted in the context of methodological limitations. In an effort to replicate the extant literature we focused on single markers that do not cover the entire region of each gene. Although the power to detect large genetic effects was acceptable, it was quite low for smaller, more realistic, effect sizes. Thus, variation across the entire gene in samples of children large enough to detect small effects have yet to be conducted. Studies aimed at better understanding these associations and testing new candidate genes arising from the recent genome wide association studies are needed. Because the sample sizes available at independent sites are lacking, the creation of a viable and collaborative pediatric bipolar disorder genetics network will be critical to the success of these replication studies

## Conclusion

Our study suggests that markers examined thus far in *COMT *and *SLC6A4 *are not associated with pediatric bipolar disorder and that if the val66met marker in *BDNF *is associated with pediatric bipolar disorder the magnitude of the association is much smaller than first reported.

## Abbreviations

BDNF: is an abbreviation of brain-derived neurotrophicfactor gene; SLC6A4: represents the serotonin transporter gene; COMT: the catechol-O-methyltransferase gene; DSM-III, -IIIR, -IV: Diagnostic and Statistics Manual of Mental Disorders Versions III, III-revised, or IV.

## Competing interests

Dr. Eric Mick receives research support from the following sources: McNeil Pediatrics, Ortho-McNeil Janssen Scientific Affairs, Pfizer, Shire Pharmaceuticals, and the National Institute of Mental Health (NIMH) and has had an advisory or consulting relationship with Pfizer, Shire Pharmaceuticals and Validus Pharmaceuticals.

Dr. Janet Wozniak receives research support from Eli Lilly and the National Institute of Mental Health (NIMH) and has a consulting relationship with the following: Pfizer, Shire Pharmaceuticals, Eli Lilly. Dr. Wozniak also serves on a speaker's bureau for both Eli Lilly and Janssen.

Dr Timothy Wilens *receives grant support from the following sources: *Abbott, McNeil, Lilly, NIH(NIDA), Merck, and Shire; is a *speaker for the following speaker's bureaus: *Lilly, McNeil, Novartis, and Shire; and is a consultant for Abbott, McNeil, Lilly, NIH (NIDA), Novartis, Merck, Shire.

*Dr. Joseph Biederman is currently receiving research support from the following sources: *Alza, AstraZeneca, Bristol Myers Squibb, Eli Lilly and Co., Janssen Pharmaceuticals Inc., McNeil, Merck, Organon, Otsuka, Shire, NIMH, and NICHD Dr. Joseph Biederman is currently a consultant/advisory board member for the following pharmaceutical companies: Janssen, McNeil, Novartis, and Shire. Dr. Joseph Biederman is currently a speaker for the following speaker's bureaus: Janssen, McNeil, Novartis, Shire, and UCB Pharma, Inc. In previous years, Dr. Joseph Biederman received research support, consultation fees, or speaker's fees for/from the following additional sources: Abbott, AstraZeneca, Celltech, Cephalon, Eli Lilly and Co., Esai, Forest, Glaxo, Gliatech, NARSAD, NIDA, New River, Novartis, Noven, Neurosearch, Pfizer, Pharmacia, The Prechter Foundation, The Stanley Foundation, and Wyeth.

Dr. Faraone receives research support from and consults to Shire Pharmaceutical Development. He receives grant support from Eli Lilly and the National Institutes of Health.

## Authors' contributions

All authors were involved in the design and execution of this study and contributed to the scientific content of this report. EM performed all statistical analyses and takes primary responsibility for the final report.

## Pre-publication history

The pre-publication history for this paper can be accessed here:


